# Self-image and 12-month outcome in females with eating disorders: extending previous findings

**DOI:** 10.1186/s40337-019-0247-1

**Published:** 2019-05-03

**Authors:** Emma Forsén Mantilla, Claes Norring, Andreas Birgegård

**Affiliations:** Department of Clinical Neuroscience, Karolinska Institute, and Center for Psychiatry Research, Stockholm County Council, Norra Stationsgatan 69, SE-11364 Stockholm, Sweden

**Keywords:** Eating disorder, Outcome, Prediction, Self-image

## Abstract

**Background:**

The interpersonal Structural Analysis of Social Behavior (SASB) model of self-image has repeatedly proven valuable in relation to eating disorder (ED) symptoms and in predicting ED outcome.

**Objective:**

We studied the association between initial self-image according to the SASB and 12-month outcome, in five diagnostic groups of female ED patients. Based on previous findings, we expected autonomy related variables (self-control/autonomy) would strongly predict outcome in anorexia nervosa (AN) groups, whereas variables related to affiliation (self-attack/love) would moderately predict outcome in bulimia nervosa (BN).

**Method:**

Participants were adult female patients, of whom 457 had AN restrictive type, 228 AN binge/purge subtype, 861 BN, 505 other specified ED and 170 binge eating disorder. Data came from the Stepwise clinical database in Sweden. Outcomes were presence/absence of ED diagnosis and self-rated ED symptoms, and we controlled for baseline ED pathology, BMI, age and general psychiatric symptoms.

**Results:**

Regression analyses showed that although the pattern differed somewhat between diagnostic groups, high initial self-love and low self-attack/self-blame predicted a more positive 12-month outcome. In some groups (AN/R in particular), these variables remained important even when baseline pathology and age were included in the analyses.

**Discussion:**

Self-image aspects once again display substantial power in predicting outcome in EDs. In AN/R patients, self-love plays an almost as crucial a role as baseline ED pathology in relation to 12-month outcome.

## Plain English summary

In eating disorders, research has found that self-image, or the way a person treats him- or herself, is important for becoming, remaining, or ceasing to be ill. This study looked at whether eating disorder patients’ self-image at the start of treatment could inform about how successful treatment was likely to be at a second measurement after 12 months. A large sample of 2221 patients from a clinical database participated, with various types of eating disorder diagnosis. We found that most important for a good outcome were high self-love and low self-attack and self-blame. There were some differences between diagnoses but the basic pattern was fairly consistent. We conclude that self-image is important and informative for the outcome of eating disorders, and that for some patients in particular (who have anorexia nervosa, especially of the restrictive subtype), self-image may be of central importance even compared to eating disorder symptoms themselves.

## Background

**Fig. 1:**
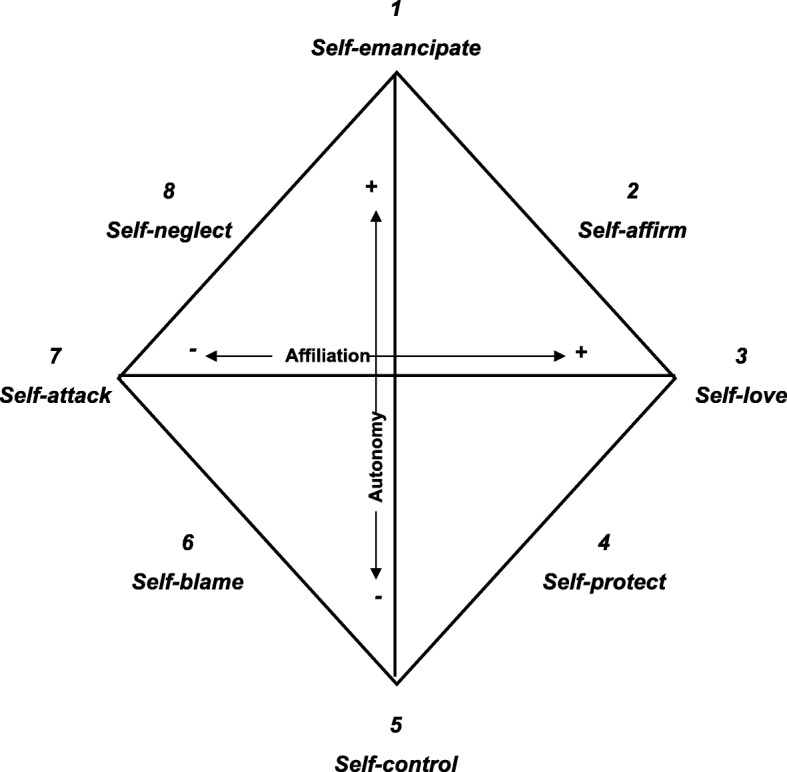
Structural Analysis of Social Behavior (SASB) Introject cluster model. The endpoints and combinations of the dimensions affiliation and autonomy form eight clusters. The clusters in the top half (Clusters 8, 1, and 2) concern autonomy, the clusters in the bottom half (6, 5, and 4) concern negative autonomy, i.e., control, and Clusters 7 and 3 are neutral in terms of autonomy. Similarly, clusters in the left half (6, 7, and 8) concern negative behaviors, the right half (2, 3, and 4) concerns positive ones, and Clusters 1 and 5 are neutral in terms of affiliation. From: Benjamin LS. Interpersonal Diagnosis and Treatment of Personality Disorders, 2nd ed. New York: The Guilford Press, 1996

In order to tailor treatment efforts, predictors of treatment outcome in the different eating disorders (ED) need to be studied. In recent years, psychological and interpersonal functioning have attracted increasing attention as potential risk- and/or maintaining factors in ED [1–3]. Self-esteem is commonly researched in relation to ED, and findings suggest that high and low self-esteem decreases vs. increases risk of ED, respectively [4–6]. The idea that interpersonal difficulties may influence the development and/or maintenance of ED via an adverse effect on self-esteem [5] has gained partial support [7]. However, global self-esteem concerns only the evaluative component of self-worth, and higher specificity may be attained using more multifaceted conceptualizations of self-image. The interpersonal Structural Analysis of Social Behavior (SASB) self-image or *introject* captures both the evaluative component of self-esteem and self-directed actions [8], i.e. how one *treats* oneself as a result of interpersonal learning. It also has theoretical (and empirically supported) implications for interpersonal functioning: the self-image forms in early interactions with attachment figures and provides a template for subsequent interpersonal behavior as people tend to behave in ways to confirm their self-image, regardless of its quality [8–10]. SASB maps self-directed behavior around two dimensions in a circumplex, where *Affiliation* (love vs. attack) constitutes the horizontal dimension and *Autonomy* (control vs. autonomy) the vertical. The dimensional end points and their combinations form eight behavior “clusters” (see Fig. 1); e.g. self-love combined with autonomy-giving form behaviors such as being spontaneous and free to explore and accept one’s feelings, needs and wishes, whereas control combined with attack form excessive adherence to both internal and external rules, and harsh self-criticism.

Previous research has shown that patients’ SASB self-image profiles are diagnostically distinct and significantly more negative than controls [11], and that negative self-image predicts poor outcome [12], treatment dropout [13] and suicidal behavior [14]. Also, we found strong associations between self-blame and inversely self-love/affirmation and ED symptoms, in several groups of female adolescent and adult ED patients, normal controls and non-treatment seeking but symptomatic women (anorexia nervosa [AN], bulimia nervosa [BN] and ED not otherwise specified [EDNOS]; [15, 16]. In adult BN patients, self-attack was also positively associated with ED symptoms.

In a study on outcome prediction of self-image among adult women with AN and BN, degree of self-love/self-attack moderately predicted outcome after three years in BN, whereas self-control powerfully predicted three year outcome in AN [17]. Adding baseline clinical characteristics in a second set of analyses, SASB overall had substantial impact on outcome over and above these. Predicting outcome from SASB alone tests whether initial self-image can be used as a prognostic tool and may suggest treatment implications connected to the SASB interpersonal model. It does not, however, exclude an influence of for example initial symptoms that affects theoretical interpretations. Adding clinically relevant baseline characteristics evaluates if the predictive power of SASB may be better explained statistically by such variables, but keeping in mind that they provide less clear indications for treatment course and for how to interact with patients in therapy than SASB variables do.

In summary, the SASB affiliation dimension seems important in BN for both initial symptom levels and outcome. For AN, self-blame and self-love/acceptance seem important for initial symptom levels, but for long-term outcome self-control was a stronger predictor. These associations need to be investigated using a shorter time-span to follow-up, and more importantly extended to include the ED diagnostic spectrum relevant to DSM-5 [18]. Examining a shorter time-span to follow up may give indications about which self-image aspects to focus for short-term treatment gains, thus complementing the long-term focus of the previous study. Including the entire ED diagnostic spectrum, as well as AN subgroups, is essential as previous research suggests that the pattern of associations may differ depending on symptom presentation [11, 16; 17]. This could have importance for future research regarding diagnostic classification, treatment interventions and outcome prediction.

### The present study

The aim was to extend Birgegård, et al.’s [17] findings through investigating the predictive value of SASB self-image on 12-month outcome in DSM-5 ED, in a new dataset. We therefore re-categorized data from patients diagnosed with the DSM-IV into five groups more similar to the diagnostic categorization in DSM-5 (AN restrictive subtype [AN/R], AN binge/purge subtype [AN/BP], BN, Other specified feeding and eating disorder (OSFED, reminiscent of EDNOS in DSM-IV), and binge-eating disorder [BED]); in Birgegård et al. [17], no subgrouping of AN was made and this is therefore an extension of that study. As in the previous study, we decided to focus on adult women (≥ 18 years) only. In the present study we were interested in the clinical utility of self-image for outcome prediction, hence keeping the study design as naturalistic as possible and maximizing ecological validity was of primary importance. Therefore, unlike the Birgegård et al. study [17], we included all ED patients available at follow-up rather than focusing patients with stable diagnoses only, and we also decided to include participants who dropped out of treatment before follow-up if they nevertheless had follow-up data. All participants received some sort of specialist ED treatment during the year preceding follow-up, but the length, magnitude and content of such treatment was not controlled for. In this way, we studied the course for these groups one year after they have entered specialist ED care in a general sense.

If self-image aspects have differential predictive value depending on diagnosis, and this can be demonstrated already at one-year follow-up, it would add to the evidence for the clinical utility of SASB self-image. We also wanted to test whether baseline scores of relevant variables (BMI, age, baseline ED pathology, overall psychiatric symptoms and baseline clinical impairment) predicted outcome and whether SASB captured additional outcome variance.

### Hypotheses

Consistent with previous findings, we hypothesized that autonomy-related variables (self-control, self-blame and inversely self-acceptance) would strongly predict outcome in AN, whereas variables related to affiliation (self-love/attack) would moderately predict outcome in BN. We did not have specific hypotheses about AN subgroups, OSFED, or BED, as there is no previous research regarding which self-image aspects may relate to outcome in these groups.

## Method

### Participants

Data came from the Stepwise clinical database, an Internet-based data collection system for specialized ED care in Sweden in use since 2005 [19]. Criteria for inclusion are medical- or self-referral to one of the participating treatment units (*N* = 38), a diagnosed DSM-IV ED, and intention to treat the patient. At the time of data extraction (29th of January, 2016), complete data from initial registration were available for 9064 adult (≥18 years) patients. Out of those, 5704 were excluded due to missing data (5383 due to missing or incomplete 12-month follow-up data,^1^ 13 due to missing data on initial ED diagnosis, 222 due to missing initial self-report data, and 86 whose diagnostic status at follow up was unclear). Also, males (*n* = 299) were excluded, as were patients who did not consent to research participation (*n* = 353). Finally, we decided to exclude two diagnostic groups. Patients diagnosed with “EDNOS Other” (*n* = 419) were excluded due to the unspecific nature of the diagnosis (also this group is unlikely to belong to the ED population; [20]), and patients diagnosed with “EDNOS type 5” (chewing and spitting) (*n* = 68) due to being arguably unrepresentative of the general ED population and few in number, i.e. < 2% of the database. The remaining sample consisted of 2221 female patients aged 18–70 (*M* = 26.3 *SD* = 8.37). Diagnostic distribution was AN/R = 457, AN/BP = 228, BN = 861, OSFED = 505, and BED = 170. Patients who dropped out of treatment but who nevertheless were followed up were not excluded from analyses. The study is clinical registry study, and the Stockholm regional ethical review board has approved the study (Dnr 2007/39–31/2).

### Instruments

*Structural Analysis of Social Behavior (SASB, 3rd surface, self-image)* assesses self-image in 36 items rated 0–100 scale with 10-point increments, yielding eight variables (“clusters”): Self-emancipation, Self-affirmation, Self-love, Self-protection, Self-control, Self-blame, Self-attack, and Self-neglect (see Fig. 1). Benjamin [21] reported acceptable internal consistency and the structure of the underlying model has been confirmed in factor analyses [21]. Armelius [22] reported an internal consistency of .87 for the Swedish version. In the present study clusters were excluded if Cronbach’s alpha was < .65 in any of the ED groups. Acceptable alphas were obtained for all clusters except cluster 1, self-emancipation.

*Eating Disorders Examination Questionnaire (EDE-Q)* [23]*.* The 36-item EDE-Q contains four subscales: restraint, eating concern, shape concern, and weight concern, and an average global score. The EDE-Q has shown good reliability and validity [24, 25]. For this study, the global score was used as a measure of overall ED pathology.

*Comprehensive Psychiatric Rating Scale – Self-rated Affective subscales* (*CPRS-S-A)* [26] is a 19-item questionnaire measuring depression, anxiety and compulsiveness. Because of the high intercorrelation between the three scales (*r* = .78–.86 in present data), we used a total score (averaging *z*-standardized scores of the three scales) as a measure of overall psychiatric symptoms. The CPRS-S-A has shown good reliability and validity [26].

*Clinical Impairment Assessment questionnaire (CIA)* [27] is a 16-item questionnaire covering impairment in domains of life typically affected by an ED: mood, cognitive functioning, interpersonal functioning, work performance and self-perception. It focuses the past 28 days and provides a single index of severity of psychosocial impairment secondary to ED features. The CIA has good psychometric properties [27].

*Structured Eating Disorder Interview (SEDI)*, developed for the Stepwise system, is a semi-structured interview used to establish DSM-IV ED diagnosis and subtype. The interview consists of between 20 and 30 questions depending on skip-rules and takes less than 20 min. Preliminary evidence shows substantial concordance between the SEDI and the Eating Disorders Examination interview concerning ED diagnosis; Kendall’s tau-b = .69, *p* < .001 [28]. Data from the SEDI was used to re-categorize patients into the five diagnostic groups in accordance with DSM-5: AN/R (including EDNOS example 1; amenorrhea absent), AN/BP (including EDNOS example 1), BN (including EDNOS example 3; lower frequency/duration of bingeing and compensation), OSFED (including EDNOS example 2, AN except not underweight, and EDNOS example 4; compensation without bingeing, but excluding EDNOS example 5; chewing and spitting, a small and atypical group, and EDNOS “other”, previously shown to be subclinical [20], and BED (EDNOS example 6). Thus our OSFED group fairly well approximates the DSM-5 text.

### Procedure

Patients were assessed by ED specialists using Stepwise prior to treatment, usually within the first three visits to the unit. After a brief orienting interview, the assessment starts with the Structured Clinical Interview for DSM-IV Axis I disorders [29] followed by the SEDI, clinical ratings, and collection of demographic and psychiatric history. After this, the patients complete self-report measures (including the EDE-Q, CPRS-S-A, CIA and SASB) on the computer. The entire assessment takes slightly more than 1 h on average.

12-month follow-up assessments were made within an 8-week window (±4 weeks) around the one-year day, and were identical to the initial assessments for all measures included here. If necessary, interviews were done by phone, and self-ratings by patients logging in to the Stepwise system from home.

### Statistical analysis

Outcome at 12 months was measured as presence/absence of ED diagnosis and levels of ED symptomatology (EDE-Q total score at 12 months). In a first set of analyses, the predictor variables were the seven SASB clusters at initial presentation alone. We also wanted to test for possible effects of baseline clinical characteristics: in a second set of analyses, we added age and baseline clinical data: BMI, CPRS-S-A, EDE-Q and CIA, as predictors alongside the seven included SASB clusters. When the outcome was EDE-Q at 12-months, separate stepwise regression models were conducted for each diagnostic group, with SASB clusters as independent in a first set of analyses and SASB as well as age and clinical scores in a second set. When the outcome variable was presence/absence of ED at 12 months, logistic regression models (with Backward LR elimination) were conducted.

## Results

### Self-image and outcome

Stepwise regressions and logistic regressions were conducted using the seven SASB clusters as predictors of outcome in the different diagnostic groups.

### ED diagnosis

For AN/R more initial Self-love, and less initial Self-affirmation and Self-attack, predicted a more positive diagnostic outcome at 12 months and in total the model explained 13% of the variance (Table 1). For AN/BP, lower Self-neglect predicted absence of diagnosis, and for BN, lower Self-attack did. In the OSFED group, lower Self-blame predicted absence of diagnosis, and in BED, no SASB variables predicted presence/absence of diagnosis at 12 months.

**Table 1:** Separate logistic regression models for the diagnostic groups with SASB self-image aspects as predictors of 12-month diagnostic status

Diagnostic group	Predictors^a^	*B (e* ^*β*^ *)*	Wald’s *X*^*2*^	*p*	Nagelkerke *r* ^*2*^
AN/R	Self-love	−.039 (.962)	15.421	<.001	
	Self-affirmation	.022 (1.022)	6.454	.011	
	Self-attack	.015 (1.015)	7.632	.006	.13
AN/BP	Self-neglect	.018 (1.018)	4.741	.029	.10
BN	Self-attack	.010 (1.010)	6.387	.011	.04
OSFED	Self-blame	.015 (1.015)	12.826	<.001	.04
BED	–				

^a^The predictor variable has to contribute ≥1% unique variance to the model in order to be reported

*Note: SASB* = Structural analysis of social behavior, *ED* = eating disorders, *AN/R* = anorexia nervosa restrictive subtype, *AN/BP* = anorexia nervosa binge-purge subtype, *BN* = bulimia nervosa*, OSFED* = other specified feeding and eating disorders, *BED* = binge eating disorder

### EDE-Q global score

Higher Self-love and lower Self-blame were the strongest predictors of positive outcome on EDE-Q in AN/R (Table 2). For AN/BP and BN, lower Self-attack and higher Self-love predicted better outcome. In OSFED, higher Self-love and lower Self-blame and Self-control predicted better outcome, and for BED, higher Self-love did so.

**Table 2:** Separate stepwise regression models for the diagnostic groups with SASB self-image aspects as predictors of 12-month EDE-Q global score

Outcome variable	Diagnostic group	Predictors^a^	*β*	*t*	*p*	*r* ^*2*^_*cum*_
EDE-Q	AN/R	Self-love	−.300	−5.551	<.001	.17
		Self-blame	.175	3.241	.001	.18
	AN/BP	Self-attack	.290	3.811	<.001	.14
		Self-love	−.150	−1.971	.035	.16
	BN	Self-attack	.185	4.615	<.001	.08
		Self-love	−.164	− 4.082	<.001	.10
	OSFED	Self-love	−.219	−4.168	<.001	.10
		Self-blame	.155	2.879	<.001	.12
		Self-control	.116	2.681	.008	.13
	BED	Self-love	−.291	−3.937	<.001	.08

^a^The predictor variable has to contribute ≥1% unique variance to the model in order to be reported

*Note: SASB* = Structural analysis of social behavior, *EDE-Q* = eating disorder examination questionnaire, *AN/R* = anorexia nervosa restrictive subtype, *AN/BP* = anorexia nervosa binge-purge subtype, *BN* = bulimia nervosa*, OSFED* = other specified feeding and eating disorders, *BED* = binge eating disorder

#### Self-image, baseline pathology, age and outcome

In a second step, we re-ran the analyses adding baseline clinical variables (BMI, EDE-Q, CIA, CPRS-S-A) and age as predictors alongside the SASB clusters.

### ED diagnosis

Absence of ED diagnosis at 12 months in patients with AN/R was associated with higher initial BMI, and lower EDE-Q and age, as well as higher SASB Self-love and lower Self-affirmation (Table 3). For AN/BP, higher BMI and lower age and CIA scores predicted a better outcome. SASB Self-control also contributed significantly, with high initial Self-control predicting a better outcome. For BN patients, lower psychiatric symptoms (CPRS-S-A) and EDE-Q and higher BMI were associated with remission at follow-up. In OSFED, lower CPRS-S-A predicted remission, and in BED, low EDE-Q and younger age did. Thus, only the AN models contained SASB self-image variables over and above baseline clinical variables and age.

**Table 3:** Separate logistic regression models for the diagnostic groups with baseline pathology^b^ and age included alongside the SASB self-image aspects as predictors of diagnostic status at 12 months

Diagnostic group	Predictors^a^	*B (e* ^*β*^ *)*	Wald’s *X*^*2*^	*p*	Nagelkerke *r* ^*2*^
AN/R	EDE-Q	.279 (1.322)	8.579	.003	
	BMI	−.109 (.897)	5.602	.018	
	Age	.038 (1.039)	6.909	.009	
	Self-love	−.035 (.965)	12.370	<.001	
	Self-affirmation	.025 (1.026)	7.856	.005	.18
AN/BP	BMI	−.134 (.874)	7.270	.007	
	Age	.047 (1.048)	4.719	.030	
	CIA	.064 (1.066)	17.169	<.001	
	Self-control	−.021 (.980)	5.572	.018	.19
BN	CPRS-S-A	.253 (1.288)	8.007	.005	
	EDE-Q	.315 (1.371)	13.419	<.001	
	BMI	−.038 (.963)	4.752	.029	.07
OSFED	CPRS-S-A	.452 (1.572)	20.024	<.001	.06
BED	EDE-Q	.357 (1.429)	3.632	.057	
	Age	.036 (1.037)	6.025	.014	.09

^a^The predictor variable has to contribute ≥1% unique variance to the model in order to be reported

^b^*Baseline pathology* = initial BMI, EDE-Q (eating disorder examination questionnaire), CIA (clinical impairment assessment) and CPRS-S-A (clinical psychiatric rating scale affective subscales) scores

*Note: SASB* = Structural analysis of social behavior, *ED* = eating disorders, *AN/R* = anorexia nervosa restrictive subtype, *AN/BP* = anorexia nervosa binge-purge subtype, *BN* = bulimia nervosa*, OSFED* = other specified feeding and eating disorders, *BED* = binge eating disorder

### EDE-Q global score

In all diagnostic groups, baseline EDE-Q predicted follow-up EDE-Q (Table 4). Variables contributing beyond this were AN/R Self-love (higher = better), AN/BP Self-attack and age (lower = better), BN Self-neglect and age (lower = better), OSFED CPRS-S-A (lower = better), and in BED, lower age and higher Self-love also predicted positive outcome.

**Table 4:** Separate stepwise regression models for the diagnostic groups with baseline pathology^b^ and age included alongside the SASB self-image aspects as predictors of EDE-Q global score at 12 months

Outcome variable	Diagnostic group	Predictors^a^	*β*	*t*	*p*	*r* ^*2*^_*cum*_
EDE-Q	AN/R	EDE-Q	.433	9.574	<.001	.28
		Self-love	−.191	−4.221	<.001	.31
	AN/BP	EDE-Q	.362	5.321	<.001	.21
		Self-attack	.184	2.714	.007	.24
		Age	.146	2.528	.012	.26
	BN	EDE-Q	.365	11.243	<.001	.17
		Self-neglect	.144	4.438	<.001	.19
		Age	.102	3.337	.001	.20
	OSFED	EDE-Q	.403	8.605	<.001	.23
		CPRS-S-A	.144	3.073	.002	.25
	BED	EDE-Q	.410	4.141	<.001	.14
		Self-love	−.187	−2.574	.011	.17
		Age	.179	2.573	.011	.21

^a^The predictor variable has to contribute ≥1% unique variance to the model in order to be reported

^b^*Baseline pathology* = initial BMI, EDE-Q (eating disorder examination questionnaire), CIA (clinical impairment assessment) and CPRS-S-A (clinical psychiatric rating scale affective subscales) scores

*Note: SASB* = Structural analysis of social behavior, *EDE-Q* = eating disorder examination questionnaire, *AN/R* = anorexia nervosa restrictive subtype, *AN/BP* = anorexia nervosa binge-purge subtype, *BN* = bulimia nervosa*, OSFED* = eating disorders not otherwise specified, *BED* = binge eating disorder

## Discussion

The study tested whether aspects of self-image as measured by the SASB could predict one-year outcome in different groups of ED patients in a similar fashion as previously [17] but with the addition of OSFED and BED, and with a shorter time to outcome. The aim was to learn more about the SASB variables predictive value alone, as well as over and above relevant baseline clinical characteristics and age, in groups closely approximating DSM-5 ED. Hypotheses that SASB autonomy-related variables would strongly predict outcome in AN, and affiliation variables would moderately predict outcome in BN, were partially supported. Affiliation variables (Self-love, Self-attack and Self-neglect) were recurrent moderate predictors in BN and explained variance was consistently higher in AN. However, rather than the autonomy dimension, affiliation was prominent in the AN groups. Unlike the Birgegård et al. [17] study, Self-control did not enter any of the models without baseline. Self-control did enter one AN/BP model but as a positive predictor, as opposed to being associated with negative 3-year outcome in AN in Birgegård et al. [17]. For OSFED and BED also, affiliation was most often involved, although the autonomy dimension did appear in OSFED models.

### Implications of self-image for 12-month outcome in the different diagnostic groups

*AN/R.* AN/R patients with less severe baseline ED, younger age, and higher Self-love, do better after 1 year in specialist care. In a previous study [16] high Self-love was strongly associated with lower ED symptom levels in 19–25 year old women with AN at presentation, consistent with the present data. An intriguing finding was that higher Self-affirmation was associated with still having an ED at 12 months. Perhaps accepting oneself “as is” when ill, allowing the self to remain static and following current impulses, is detrimental even though the SASB variable is positive at face value. Thus, the “anorexic self” rather than the “authentic self” may be reflected here, and may engender resistance to change [30]. Similarly, the positive variable Self-protection predicted poorer outcome after three years in AN in the Birgegård et al. [17] study, implying that a wish to protect and preserve the self as is when ill hinders improvement over three years. These associations seem unique to AN in these data, and may be understood in light of research indicating denial of illness and resistance to treatment as typical of AN [31, 32].

According to SASB theory, affirming and accepting the patient (corresponding to self-affirmation) is the typical behavior to elicit self-disclosure and willingness to explore the self. The speculation presented here would suggest, similar to the argument in Birgegård et al. [17], that this be done firmly within a context of establishing what aspects of self-treatment are consistent with the ED and which represent distancing from it, so as not to inadvertently affirm a stance that maintains the disorder. In therapy it may be useful to externalize the illness, examining both its advantages and disadvantages, much in line with for example motivational interviewing.

*AN/BP.* Based on SASB data alone (excluding baseline pathology and age), Self-neglect, Self-attack, and Self-love conveyed information regarding outcome for AN/BP. Including all variables, positive outcome was associated with higher BMI and Self-control, and lower Self-attack, age and ED symptoms. Interestingly, the effect of Self-control was opposite the one in Birgegård et al. [17], where it predicted poorer outcome over 3 years. Perhaps Self-control may enable self-discipline and adherence to treatment over the short term, especially in AN/BP where binge eating and/or purging are present and inhibitory control is a prominent deficit (e.g. [33, 34]. Over time however, it may maintain symptoms or convey vulnerability for relapse, to which this group appears especially susceptible [35]. This is reminiscent of findings that high persistence and constraint are typical of AN [36], and that impulsivity is related to long-term recovery from AN [37], interpreted in that study as a trait that “tempers the rigidity and intractability often associated with AN” (p. 977). Importantly, this raises the possibility that treatment recruiting self-control in the service of behavior change may inadvertently maintain vulnerability, as was also speculated in Birgegård et al. [17].

*BN.* BN patients with lower general psychiatric and ED symptoms and higher BMI are more likely to remit, and self-rated symptoms are likely to improve if they are younger and have lower Self-neglect. When SASB alone predicted outcome, Self-attack and Self-love explained variance, consistent with previous studies demonstrating the importance of the affiliation dimension for this group [11, 12, 17]. An interesting finding was the impact of Self-neglect when adding baseline. Self-neglect is consistent with negative impulsivity, present in the central behavioral symptoms of BN. This may suggest that the specific addition in SASB of letting go of the self in a negative way conveys information not contained in baseline clinical data. In patients with high Self-attack and Self-neglect, it may be helpful to specifically train self-compassion, which has shown promise in the treatment of borderline personality disorder [38], borderline being common in BN [39], or emotion regulation skills, such as in integrative cognitive-affective therapy for BN (ICAT-BN; [40]).

#### OSFED

With SASB alone, Self-blame was important to diagnosis, and Self-love, Self-blame and Self-control were important to ED symptoms, but these did not remain after inclusion of baseline clinical characteristics. OSFED is more heterogeneous due to the unspecific and broad nature of this category, and therefore a consistent pattern of variables predicting outcome might be less likely to occur. Subgroups of OSFED may have more distinct mechanisms and vulnerabilities, which may be elucidated in future research.

#### BED

SASB Self-love contributed to EDE-Q outcome whether or not covariates were included. The affiliation axis approximates self-esteem [41], and there is evidence for low self-esteem predicting onset of binge eating among dieters [42]. Individuals with BED display significantly lower levels of self-esteem compared to normal and obese individuals, which in turn is associated with higher ED pathology [43]. Also, health care professionals tend to hold negative attitudes regarding obesity [44, 45], which may cause therapists to subtly reinforce, or less effectively treat, negative self-views in these patients. In addition, Brandsma [46] found that obese patients overestimated their physicians’ negative attitudes towards them, which is consistent with a negative self-image in SASB theory.

### General discussion: similarities and differences in relation to previous research

These results confirm the capacity of SASB self-image to predict outcome in ED even at a short time interval. Compared to previous findings [17], our findings regarding AN were not as clearly related to self-control. Instead, the affiliation dimension was prominent with some addition of self-control in AN/BP and OSFED. When baseline pathology and age were included as predictors, such variables entered all models but in six models, SASB added variance. Self-blame never entered alongside baseline pathology, indicating that similar variance is involved. In Forsén Mantilla et al. [16], self-blame was associated with concurrent ED symptoms in all ED diagnoses.

In contrast to the present study, the Birgegård et al. [17] study restricted analyses to patients with stable diagnoses across the first 18 months in an attempt to specifically target typical psychological profiles in the diagnostic groups. To control for the possibility that this difference influenced results, we reran our analyses in the subsample of patients with stable diagnoses (or remission, i.e. no diagnostic drift) over the follow-up year (*n* = 1646, 74.1% of the original sample). The pattern of results closely resembled that obtained in the whole sample and does not alter conclusions about similarity between the studies (data not shown).

Also, although the targeted constructs were similar, different measures were used in the present study (except SASB), which may help explain differences in results. For example, younger age, a variable not included in the previous study, was associated with better prognosis in several models, suggesting shorter illness duration and associated increased chance for better outcome [47]. Another possibility, as noted, is that variable impact changes over time. SASB self-image is a trait-by-situation measure affected by both stable and transient factors [48]. Thus, short-term, self-control may enable treatment adherence and behavior change, but long-term the same trait may induce vulnerability to relapse, analogous perhaps to a high capacity to withstand work-related stress, which may be positive short-term but confer risk of negative outcomes over longer periods. Affiliation axis findings in BN are similar to Birgegård et al. [17] suggesting pervasive and stable impact of the Self-attack - Self-love dimension.

A third difference in this study was the attempt to include the whole ED diagnostic spectrum as it is represented in DSM-5. This entailed using AN subtypes, as well as including previous EDNOS patients in AN and BN. Regarding diagnostic groupings however, it is possible (perhaps even likely) that diagnostic boundaries map imperfectly onto pathogenic mechanisms or vulnerability types. For example, a subgroup of restrictive patients not defined in the DSM may be especially characterized by vulnerability related to self-control. If our failure to find negative prediction from Self-control as in Birgegård et al. (2009) is seen as a non-replication rather than interpreted along the lines we have suggested above, inconsistent results across studies may stem from case mix differences obscured by DSM groupings. Other approaches to describing patients based on central pathogenic mechanisms (for example the Research Doman Criteria project, RDoC; https://www.nimh.nih.gov/research-priorities/rdoc/index.shtml) may provide a more accurate framework for research, within which more consistent results may be found.

### Limitations

A primary limitation is attrition; only 60% of the complete patient sample had the relevant follow-up data. As noted however, few and minimal differences existed between the included group and the attrition group. It is nonetheless possible that outcome prediction might have been different if the entire initial sample had been used.

Another limitation is the fact that we have attempted to construct the DSM-5 diagnostic categories based on DSM-IV diagnostic data. We have no reason to believe that the diagnostic categories would look very different from ours if they had been the result of using an instrument specifically designed to capture the DSM-5 diagnoses. Nevertheless, we could not perfectly reproduce DSM-5 diagnoses, which may have affected our diagnostic distribution.

## Conclusion

Self-image aspects may provide clinically useful information at the beginning of treatment, especially in AN, since self-image consistently and strongly predicts outcome in these patients. However, further clarification of the role of self-control in AN over different time periods is needed.

## Footnotes

1 Some of these had not yet reached deadline for follow-up. Out of those who were possible to follow-up, 60% had complete follow-up data. When comparing baseline data from patients who had follow-up data with those who did not, there were some differences that reached significance due to the large *N* (the final sample was older, higher in self-control and lower on self-neglect) but the effect sizes were minimal (data not shown).
